# Separation, Purification, Structural Characterization, and In Vitro Hypoglycemic Activity of Polysaccharides from *Panax notoginseng* Leaves

**DOI:** 10.3390/molecules30040830

**Published:** 2025-02-11

**Authors:** Xueling Zhang, Chongying Shi, Zilin Wang, Jiahe Dai, Chunhua Guan, Jun Sheng, Liang Tao, Yang Tian

**Affiliations:** 1 College of Food Science and Technology, Yunnan Agricultural University, Kunming 650201, China; zhangxueling2025@163.com (X.Z.); shichongying@163.com (C.S.); wangzilin928@163.com (Z.W.); daijiahe11@163.com (J.D.); 13885721152@163.com (C.G.); ynau@163.com (J.S.); 2 Engineering Research Center of Development and Utilization of Food and Drug Homologous Resources, Ministry of Education, Yunnan Agricultural University, Kunming 650201, China; 3 Yunnan Key Laboratory of Precision Nutrition and Personalized Food Manufacturing, Yunnan Agricultural University, Kunming 650201, China; 4 Puer University, Puer 665000, China

**Keywords:** *Panax notoginseng* leaf polysaccharides, isolation and purification, structural characterization, antioxidant activity, hypoglycemic activity

## Abstract

This study optimized the extraction process of crude polysaccharides from *Panax notoginseng* leaves (PNLP) using the ultrasonic-assisted dual-enzyme method through a single-factor combined with response surface experiment. The crude polysaccharides were subsequently purified and isolated with DEAE-Cellulose 52, followed by structural analysis, evaluation of antioxidant activity, and examination of digestive enzyme inhibition. The hypoglycemic effects of the purified components were further clarified. The results indicated that the optimized crude polysaccharide had an extraction yield of 17.13 ± 0.29%. The purified fraction PNLP-3 (eluted with 0.3 M NaCl) was obtained through DEAE-Cellulose 52 chromatography, exhibiting a total sugar content of 81.2% and a molecular weight of 16.57 kDa. PNLP is primarily composed of arabinose, galactose, and galacturonic acid, with molar percentages of 20.24%, 33.54%, and 24.27%, respectively. PNLP-3 is mainly composed of arabinose and galactose, with molar percentages of 29.97% and 49.35%, respectively. In this study of hypoglycemic activity, the IC_50_ values of PNLP-3 for α-glucosidase and α-amylase inhibition were 1.045 mg/mL and 9.53 mg/mL, respectively. Molecular docking results confirmed that PNLP-3 exhibits better inhibitory activity against α-glucosidase. Furthermore, PNLP-3 alleviated hyperglycemia in insulin-resistant HepG2 cells by enhancing glucose consumption and glycogen synthesis. The antioxidant activity of PNLP-3 exhibited a positive correlation with its concentration, potentially contributing to its hypoglycemic effects by reducing oxidative stress. These findings underscore the therapeutic potential of *Panax notoginseng* leaf polysaccharides in managing type 2 diabetes and offer new perspectives on the use of natural polysaccharides for regulating blood glucose.

## 1. Introduction

Diabetes mellitus is a chronic metabolic condition marked by consistently high blood glucose levels, which typically result from either insulin resistance or inadequate insulin secretion [1]. This disease is commonly classified into two main types: type 1 diabetes mellitus (T1DM) and type 2 diabetes mellitus (T2DM) [2]. Among these, T2DM is the most prevalent form and is associated with a higher risk of complications, such as cardiovascular diseases, kidney damage, and nerve impairment [3]. Common treatments for T2DM are oral metformin, sulfonylurea hypoglycts, or injections of GLP-1 receptor agonists and SGLT-2 inhibitors to control blood glucose, but long-term use of such drugs can lead to liver and kidney function impairment [4,5]. Therefore, an increasing number of researchers are focusing on developing safer and more bioactive hypoglycemic agents from natural plants. Recent studies have highlighted the beneficial effects of plant-derived polysaccharides in reducing blood glucose levels. For example, *Lycium barbarum* polysaccharides [6] and *Maidong* polysaccharides [7] can play a hypoglycemic role by inhibiting digestive enzyme activity, improving insulin sensitivity and promoting insulin secretion.

*Panax notoginseng* (Burkill) F. H. Chen, a herbaceous species within the Panax genus of the Araliaceae family, is primarily distributed in the Yunnan and Guangxi provinces of China, with some cultivation occurring in the southern United States. It demonstrates considerable potential for both medicinal and dietary uses [8]. Research has demonstrated that *Panax notoginseng* leaves are abundant in polysaccharides, saponins, flavonoids, and other bioactive compounds, which have physiological effects such as anti-inflammatory, antioxidant aging, immunomodulation, neuroprotection, and hypoglycemia and hypolipidemia [9,10]. Recent research on *Panax notoginseng* leaves has highlighted the importance of efficient extraction techniques for the effective isolation of polysaccharides. Hot water extraction or acid-base extraction are traditional methods. In contrast, modern extraction techniques, such as ultrasound, microwave, and enzyme-assisted extraction, show significant potential in improving efficiency and preserving better biological activity. For instance, Wang et al. [11] reported that the polysaccharide yield obtained through the ultrasonic-assisted multi-enzyme method increased from 6.43% without enzymes to 26.68%. Wu et al. [9] compared the effects of ultrasonic disruption extraction and hot reflux extraction on the bioactivity of *Panax notoginseng* flower polysaccharides. These results showed that ultrasonic disruption extraction could effectively improve the physicochemical and functional properties, as well as the bioactivity of the polysaccharides. Simultaneously, the biological activity of polysaccharides is closely linked to their purity and structural composition. DEAE-Cellulose 52 is an ion-exchange resin commonly employed for purifying naturally derived polysaccharides owing to its high selectivity for biological macromolecules with distinct charge properties. The ion-exchange process relies on the interaction between the negatively charged groups on the polysaccharide molecules (such as sulfate or carboxyl groups) and the positively charged sites on the DEAE-Cellulose 52 matrix, making it an ideal tool for purifying complex polysaccharides [12]. Research has demonstrated that DEAE-Cellulose 52 can notably enhance both the purity and yield of polysaccharides while preserving their biological activity. In addition, this resin is also an effective method for preparing homogeneous polysaccharides [8]. Therefore, the efficient extraction and purification of polysaccharides is also an important area of research.

This study systematically optimized the process conditions for extracting PNLP using ultrasound-assisted dual-enzyme extraction and purified the extracted polysaccharides. The purified component PNLP-3 was characterized in terms of its structure, antioxidant activity, and inhibitory effects on digestive enzymes. Molecular docking simulations were performed to investigate the binding forms of the major monosaccharides in PNLP-3 with α-glucosidase and α-amylase. Finally, an IR-HepG2 cell model was established to explore the effects of PNLP-3 on glucose consumption, glycogen synthesis, and key oxidative stress enzymes in insulin-resistant cells. This study offers a theoretical foundation for the extensive development and application of *Panax notoginseng* leaf polysaccharides, highlighting their potential as natural hypoglycemic compounds and providing data support for further research on their in vivo hypoglycemic activity.

## 2. Results

### 2.1. Single-Factor Experiment Analysis

**Figure 1:**
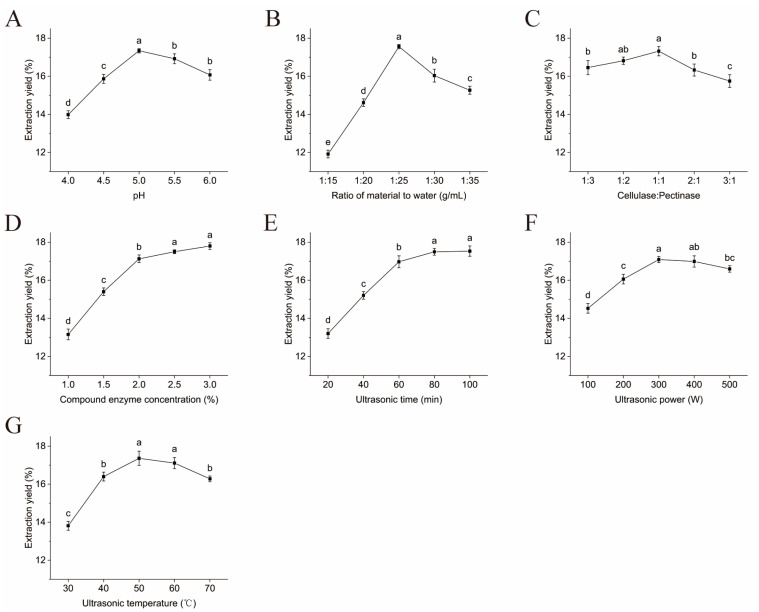
Effects of seven single factors on the polysaccharide extraction yield. (**A**) Different enzymatic hydrolysis pH values, (**B**) material/liquid ratio, (**C**) cellulase/pectinase ratio, (**D**) enzyme dosage, (**E**) ultrasonic time, (**F**) ultrasonic power, and (**G**) ultrasonic temperature. Note: The different superscript letters indicate a significant difference (*p* < 0.05) within the row based on Duncan’s test.

Figure 1 depicts the influence of seven single factors on the polysaccharide extraction yield. With the exception of enzyme dosage and ultrasound duration, all other factors exhibit a trend of initially increasing and then decreasing the extraction yield. As shown in Figure 1A,B, the polysaccharide extraction yield is highest at a pH of 5.0, where enzyme activity is optimal, thereby enhancing cell wall breakdown and facilitating polysaccharide release. The ratio of material to liquid primarily influences the extraction yield of polysaccharides by reducing solution viscosity, increasing solid-liquid contact area, and dispersing ultrasonic energy [13]. The optimal ratio of material to liquid for polysaccharide extraction is 1:25 g/mL. When the ratio of liquid to material is low, the viscosity of the extract is higher, and when the ratio is excessively high, it can disperse ultrasonic energy, both of which can lead to a decrease in the polysaccharide extraction yield. Figure 1C,D demonstrate the impact of enzymes on polysaccharide extraction yield. The primary components of the cell wall of *Panax notoginseng* leaves are cellulose and pectin. The polysaccharide extraction yield reaches a maximum of 17.32 ± 0.24% when the cellulase/pectinase ratio is 1:1. When the addition level of the composite enzyme was 2.0%, the polysaccharide extraction yield was 17.05 ± 0.21%. Increasing the enzyme concentration further did not lead to notable changes in the polysaccharide extraction yield. This may be attributed to the fact that, at an enzyme addition of 2.0%, sufficient reaction with the substrate had already occurred, aligning with the results reported by Chu et al. [14].

The mechanical vibration and cavitation effects of ultrasound assist in disrupting cell walls, promoting the release of polysaccharides from cells into the solvent [15]. As illustrated in Figure 1E, the polysaccharide extraction yield initially increases and then levels off as the ultrasonic time is extended. At 60 min, the polysaccharide extraction yield reaches 16.97 ± 0.31%. Considering energy-saving and time-cost factors, the optimal ultrasonic time is 60 min. Figure 1F,G show that the highest extraction yield was achieved at an ultrasound power of 300 W and a temperature of 50 °C. As ultrasonic power and temperature continue to increase, the cavitation and thermal effects caused by higher ultrasonic power lead to polysaccharide degradation or oxidation into other substances. Additionally, higher temperatures inhibit enzyme activity, leading to a reduction in the polysaccharide extraction yield. In conclusion, the four factors of pH, material/liquid ratio, compound enzyme addition, and ultrasonic time, which have more significant effects on the polysaccharide extraction rate, were selected for response surface optimization.

### 2.2. Response Surface Optimization Experiment

The response surface experiment was designed using Design-Expert 13.0, with the design and results presented in Table 1 and the analysis of variance provided in Table 2. The data from the 29 experiments in Table 1 were analyzed through multivariate regression, yielding the following regression equations:

*Y* = 17.18 + 0.0787*A* + 0.3550*B* + 0.6624*C* + 0.2602*D* + 0.0797*AB* + 0.2461*AC* + 0.2738*AD* − 0.0784*BC* + 0.1356*BD* − 0.0415*CD* − 0.4748*A*^2^ − 0.9275*B*^2^ − 0.5551*C*^2^ − 0.4116*D*^2^

The ANOVA results are shown in Table 2. The regression model is highly significant (*p* < 0.01), and the misfit term *p* = 0.0865 > 0.05, which is not significant, confirms the validity of the model. The model coefficient of determination, *R*^2^ = 0.9477, and the corrected coefficient of determination, *R*^2^*adj* = 0.8954, indicate that the smaller the degree of data dispersion, the higher the reliability of the fitted model. The larger the *F*-value is, the stronger the influence of each factor on the extraction rate. Based on the *F*-values, the factors affecting the polysaccharide extraction rate are ranked as follows: compound enzyme addition (*C*) > material/liquid ratio (*B*) > sonication time (*D*) > pH (*A*).

**Figure 2:**
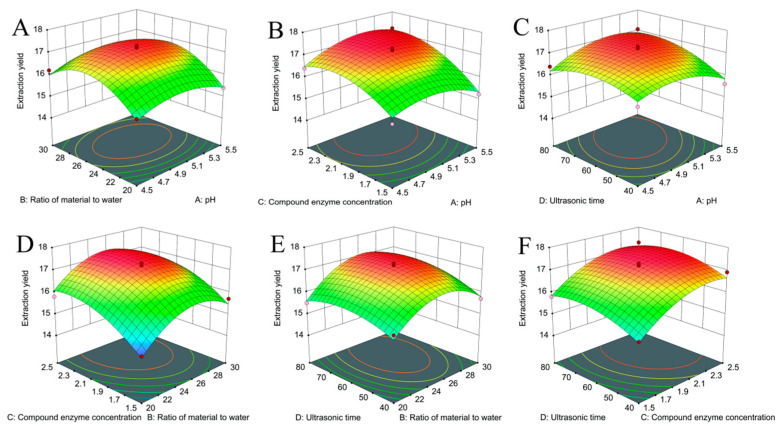
Response surface plots of the interactions between different factors. (**A**) pH and liquid/material ratio; (**B**) pH and amount of compound enzyme added; (**C**) pH and ultrasonic time; (**D**) liquid/material ratio and amount of compound enzyme added; (**E**) liquid/material ratio and ultrasonic time; (**F**) amount of compound enzyme added and ultrasonic time.

As illustrated in Figure 2, the steeper surface slopes corresponding to *B*, *C*, and *D*, accompanied by elliptical contour lines, suggest that the interactions among the material/liquid ratio, the amount of composite enzyme added, and sonication time have a significant impact on the polysaccharide extraction yield. Conversely, the gentler surface slope associated with *A* suggests a minor influence of pH on the polysaccharide extraction yield, consistent with the ANOVA results. The optimal process for polysaccharide extraction was determined by response surface optimization as follows: pH of 5.18, material/liquid ratio of 1:24.77, compound enzyme addition of 2.23%, and ultrasonic time of 57.71 min, at which the theoretical prediction of the extraction rate of PNLP was 17.32%. Considering the needs of actual production, the parameters were adjusted so the pH was 5.2, the material/liquid ratio was 1:25, the cellulase/pectinase ratio was 1:1, the addition of compound enzyme was 2.2%, the ultrasonic time was 58 min, the ultrasonic power was 300 W, and the ultrasonic temperature was 50 ℃. Three parallel experiments conducted under these adjusted conditions yielded an extraction rate of PNLP of 17.13 ± 0.29%. The minimal discrepancy between the predicted and experimental values suggests that the model accurately predicts the polysaccharide yield.

### 2.3. Purification and Chemical Composition Analysis of Panax Notoginseng Leaf Polysaccharides

**Figure 3:**
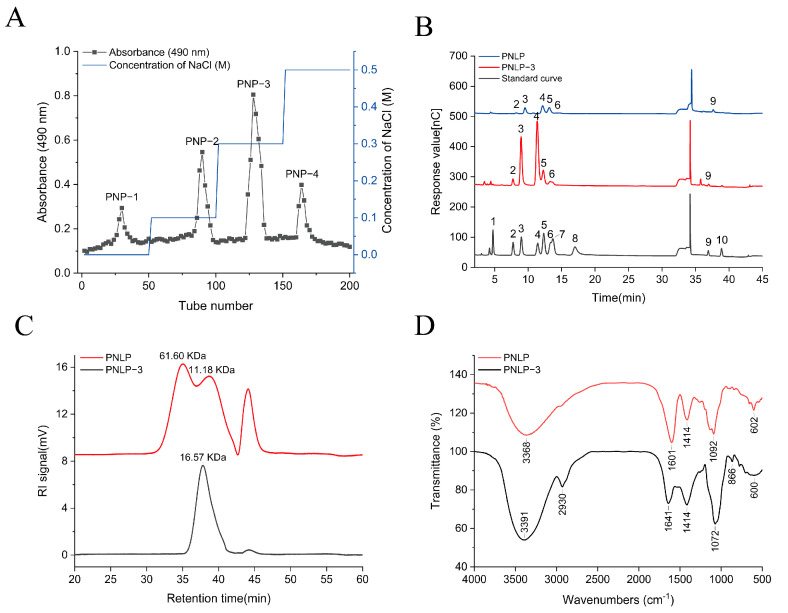
(**A**) DEAE-Cellulose 52 elution curve of PNLP and PNLP-3; (**B**) ion chromatography (1. Fuc; 2. Rha; 3. Ara; 4. Gal; 5. Glc; 6. Man; 7. Xyl; 8. Rib; 9. GalA; 10. GlcA); (**C**) HPGPC profile; and (**D**) infrared spectrum.

PNLP was purified by DEAE-Cellulose 52, yielding four different fractions: PNLP-1, PNLP-2, PNLP-3, and PNLP-4 (Figure 3A). Table 3 summarizes the yields and chemical compositions of PNLP, PNLP-1, PNLP-2, PNLP-3, and PNLP-4. After freeze-drying, the yields of the four fractions were calculated to be 0.78 ± 0.16%, 2.69 ± 0.24%, 5.64 ± 0.21%, and 1.04 ± 0.14%, respectively. The total sugar content of the four components was 59.53 ± 0.85%, 70.31 ± 0.85%, 81.20 ± 1.17%, and 74.55 ± 1.08%, respectively. PNLP-3, which exhibited the highest yield and total sugar content, was selected for further study. After purification with DEAE-Cellulose 52, the total sugar content of PNLP-3 was 81.20 ± 1.17%, an increase of 229.01%, while the protein content was 1.25 ± 0.17%, a decrease of 79.67%. This indicates that DEAE-Cellulose 52 effectively separated and purified the PNLP.

Figure 3B illustrates the monosaccharide compositions of PNLP and PNLP-3. The findings show that the monosaccharide profiles of both PNLP and PNLP-3 are similar. Both PNLP and PNLP-3 consist of six monosaccharides: rhamnose, arabinose, galactose, glucose, mannose, and galacturonic acid. The molar percentages of the monosaccharides in PNLP are 3.43%, 20.24%, 33.54%, 12.92%, 2.34%, and 24.27%, respectively. For PNLP-3, the molar percentages are 4.70%, 29.97%, 49.35%, 6.49%, 4.10%, and 2.90%, respectively. Chan et al. [16] isolated a water-soluble polysaccharide from *Panax notoginseng* predominantly composed of galactose and arabinose. Liu et al. [17] extracted a novel polysaccharide from *Panax notoginseng* residues, with galactose, arabinose, and galacturonic acid as the main components, in molar proportions of 33.3%, 25.2%, and 17.1%, respectively. Although the monosaccharide compositions across studies are comparable, slight differences in their molar ratios were observed.

### 2.4. Structural Characterization

#### 2.4.1. Molecular Weight

The purity and molecular weight of PNLP and PNLP-3 were assessed using High Performance Gel Permeation Chromatography (HPGPC). As depicted in Figure 3C, the GPC profile of PNLP exhibits an asymmetric double peak pattern, suggesting that it is a heterogeneous polysaccharide. In contrast, the GPC profile of PNLP-3 shows a symmetric single-peak structure, indicating it is a homogeneous polysaccharide with a molecular weight of 16.57 kDa, suggesting that PNLP-3 is a low-molecular-weight polysaccharide. Previous studies have indicated that low-molecular-weight polysaccharides facilitate transmembrane uptake and exhibit a higher affinity for cell receptors, leading to enhanced biological activities [18]. The results indicate that purification using DEAE-Cellulose 52 effectively separates and purifies PNLP, yielding a homogeneous polysaccharide.

#### 2.4.2. Fourier Transform Infrared Spectroscopy (FT-IR)

Infrared spectroscopy is a key technique for characterizing the structure of polysaccharides. As shown in Figure 3D, the infrared spectra of PNLP and PNLP-3 display similar peak wavelengths, with absorption bands at 3368 cm^−1^, 3391 cm^−1^, and 2930 cm^−1^, which are attributed to the stretching vibrations of the -OH and -CH groups on the sugar ring, respectively. The absorption peak of PNLP-3 at 3391 cm^−1^ shifts to a higher wavenumber, suggesting a more ordered molecular structure of the polysaccharide, which results in enhanced vibrations of the C-H or O-H bonds within the molecule [19]. The absorption peaks at 1601 cm^−1^ and 1641 cm^−1^ correspond to the stretching vibrations of the C=O bond, which are characteristic of acetyl or carboxyl ester groups, thereby confirming the presence of uronic acid in both PNLP and PNLP-3 [20]. Additionally, the absorption peak at 1414 cm^−1^ is attributed to the C-H bending vibration in carbohydrates [21]. The characteristic absorption peaks at 1092 cm^−1^ and 1072 cm^−1^ are typical of the pyranose sugar ring [22], while the peak at 600 cm^−1^ is also associated with the pyranose ring backbone [23], confirming that both PNLP and PNLP-3 are pyranose-type sugars. The distinct absorption at 866 cm^−1^ in PNLP-3 is attributed to the deformation vibration of the C-H bond in the β-configuration of the pyranose sugar ring [24], indicating that PNLP-3 predominantly consists of β-glycosidic bonds in the pyranose form.

#### 2.4.3. Scanning Electron Microscopy (SEM)

**Figure 4:**
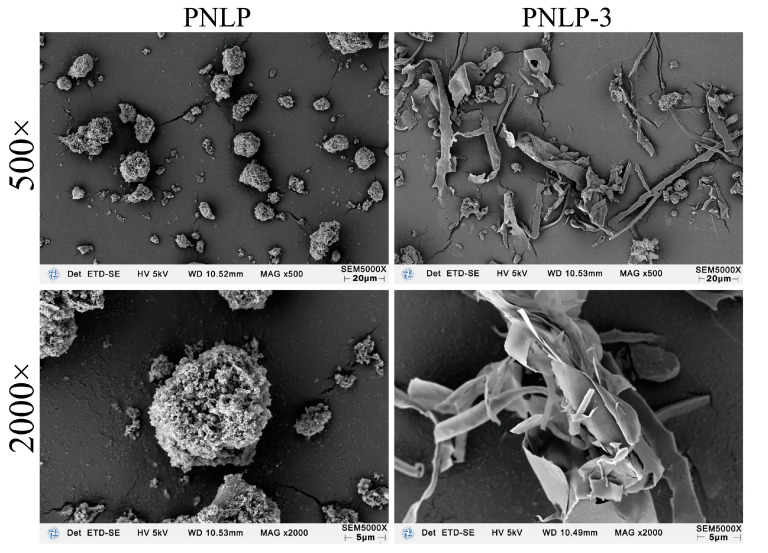
Scanning electron microscope images of PNLP and PNLP-3.

Figure 4 shows the surface morphology of PNLP and PNLP-3 at different magnifications. PNLP exhibits an aggregated, granular structure with uniform particle sizes that are well-dispersed, with significant gaps between the particles. In contrast, PNLP-3 primarily forms a flaky structure with a more scattered distribution, and the surfaces of the flakes are relatively smooth. Compared to PNLP-3, the surfaces of PNLP particles are rougher and exhibit a porous structure, which is attributed to PNLP being a coarse polysaccharide with a complex composition and uneven molecular weight [25]. The results indicate that the solid morphologies of PNLP and PNLP-3 differ, which is due to variations in their molecular weights and chemical compositions.

### 2.5. Antioxidant Activity

**Figure 5:**
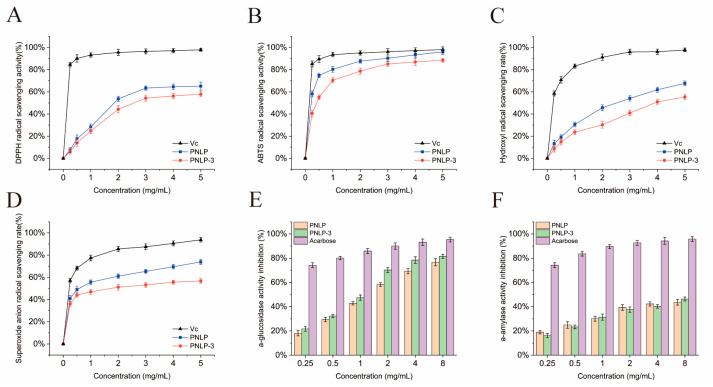
Effects of PNLP and PNLP-3 on the scavenging activities of (**A**) DPPH, (**B**) ABTS, (**C**) hydroxyl, and (**D**) superoxide anion radicals; inhibitory effects on the activities of (**E**) α-glucosidase and (**F**) α-amylase.

High levels of free radicals can destroy cell structure, triggering gene mutations promoting human diseases and the aging process [26]. The antioxidant capacity of polysaccharides serves as a key indicator for assessing their biological activities. Figure 5A–D illustrate the scavenging activity of PNLP and PNLP-3 against DPPH, ABTS, hydroxyl, and superoxide anion radicals. The radical scavenging capacity of PNLP and PNLP-3 increased progressively with increasing polysaccharide concentration, ranging from 0.25 to 5 mg/mL, following a concentration-dependent pattern. When the polysaccharide concentration was 5 mg/mL, the maximum scavenging of the four free radicals by PNLP was 65.16 ± 3.66%, 96.09 ± 2.48%, 67.60 ± 2.19%, and 73.88 ± 2.28%, respectively. The maximum scavenging of the four free radicals by PNLP-3 was 57.90 ± 2.31%, 88.50 ± 1.74%, 55.35 ± 2.30%, and 56.70 ± 2.08%, respectively. It is important to note that PNLP demonstrated superior antioxidant activity compared to PNLP-3, primarily attributed to the synergistic effect of the phenolic and flavonoid compounds present in PNLP [27]. This aligns with the results of Gao et al. [18] and Hu et al. [28], who observed that crude polysaccharides possess greater antioxidant activity than their purified counterparts. Additionally, Duan et al. [29] demonstrated that polysaccharides with lower molecular weights derived from fermentation mycelium exhibit enhanced antioxidant activity. However, although the molecular weight of PNLP-3 is lower than that of PNLP, its antioxidant activity is weaker, indicating that molecular weight is not the sole factor influencing the antioxidant activity of polysaccharides. Furthermore, the GPC profile of PNLP indicates that it is a heteropolysaccharide with a broader molecular weight distribution, allowing it to more effectively react with various types of free radicals, thereby exhibiting stronger antioxidant activity. These results suggest that *Panax notoginseng* leaf polysaccharides have significant potential as natural antioxidants in functional foods.

### 2.6. Research on In Vitro Hypoglycemic Activity

#### 2.6.1. Digestive Enzyme Inhibitory Activity

1. Enzyme Inhibition Activity

The hypoglycemic mechanisms of polysaccharides primarily involve the inhibition of digestive enzyme activity, enhancement of insulin sensitivity, and modulation of oxidative stress responses [30]. As shown in Figure 5E,F, compared to acarbose, both polysaccharides significantly inhibit the activities of α-glucosidase and α-amylase, with stronger inhibition of α-glucosidase than α-amylase. The IC_50_ values for α-glucosidase inhibition by PNLP-3 and PNLP are 1.045 mg/mL and 1.496 mg/mL, respectively, while the IC_50_ values for α-amylase inhibition are 9.53 mg/mL and 11.16 mg/mL, respectively. In summary, PNLP-3 exhibits stronger digestive enzyme inhibition than PNLP, possibly due to its higher content of uronic acid. The hydroxyl and carboxyl groups on the sugar chain may interact with the amino acid residues of the digestive enzymes [4], resulting in more effective enzyme activity inhibition. The IC_50_ value of PNLP-3 for α-glucosidase inhibition is lower than that of *moringa oleifera* leaf polysaccharides (IC_50_ = 8.02 mg/mL) reported by Gu et al. [31] and *momordica charantia* polysaccharides (IC_50_ = 4.59 mg/mL) reported by Zhang et al. [32]. The IC_50_ value of PNLP-3 for α-amylase inhibition is higher than that of *tricholoma matsutake* polysaccharides (IC_50_ = 3.75 mg/mL) reported by Yang et al. [33] and *chaenomeles speciosa* seed polysaccharides (IC_50_ = 6.24 mg/mL) reported by Deng et al. [34]. One possible explanation is that the relatively small molecular weight and shorter sugar chains of PNLP-3 allow it to more readily interact with the active site of α-glucosidase, leading to enhanced inhibitory effects. On the other hand, polysaccharides with larger molecular sizes and longer sugar chains can provide more active sites for interactions with α-amylase, thereby exerting stronger inhibition of its activity. These results suggest that DEAE-Cellulose 52 anion exchange purification can effectively enrich the uronic acid content, thereby enhancing the digestive enzyme inhibitory activity of the polysaccharides.

2. Molecular Docking

**Figure 6:**
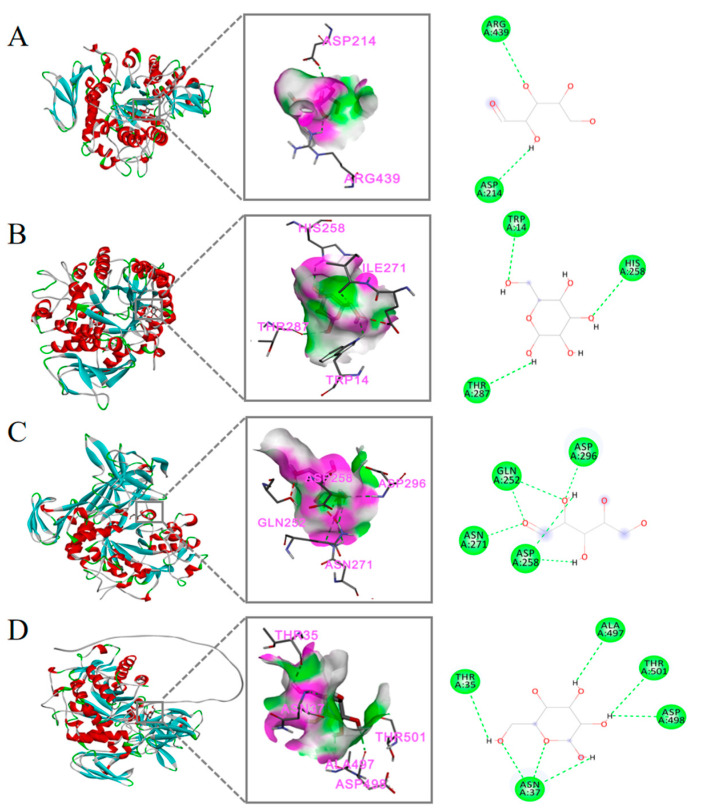
Molecular docking results: (**A**) α-glucosidase and arabinose; (**B**) α-glucosidase and galactose; (**C**) α-amylase and arabinose; (**D**) α-amylase and galactose.

Molecular docking is commonly used to predict intermolecular interactions and is a common aid in drug design and molecular function studies [35]. As shown in Figure 6, the molecular docking results show the three-dimensional interactions between arabinose and galactose with α-glucosidase and α-amylase. Docking energy values below −4.25 kcal/mol are typically regarded as indicative of some binding activity between the two, while values below −5.0 kcal/mol suggest strong binding activity [36]. α-glucosidase binds to arabinose with a −5.1 kcal/mol (Figure 6A), and α-glucosidase binds to galactose with a −5.9 kcal/mol (Figure 6B). The binding energy of α-amylase to arabinose was −4.3 kcal/mol (Figure 6C), and that of α-amylase to galactose was −5.3 kcal/mol (Figure 6D). The results showed lower binding energy and stronger interaction between α-glucosidase and polysaccharides, which aligned with the findings from the enzyme inhibitory activity assay. The findings demonstrate that α-glucosidase exhibits a lower binding affinity to the polysaccharide, leading to stronger interactions and more pronounced inhibitory effects. This is in agreement with the results from the enzyme inhibition assay. The hydroxyl (-OH) groups on the arabinose and galactose molecules form hydrogen bonds with the amino acid residues at the active site of the digestive enzymes, stabilizing the binding between the sugar molecules and the enzymes, thereby interfering with the normal catalytic function of the enzymes [37]. The monosaccharide composition revealed that PNLP-3 contains more arabinose and galactose, which contributes to its stronger α-glucosidase and α-amylase inhibition compared to PNLP, consistent with the enzyme inhibition assay results. These findings further suggest that PNLP-3 holds significant potential for hypoglycemic effects.

#### 2.6.2. PNLP-3 Regulates IR-HepG2 Cell Hypoglycemic Assay

(1) MTT Toxicity Assay

**Figure 7:**
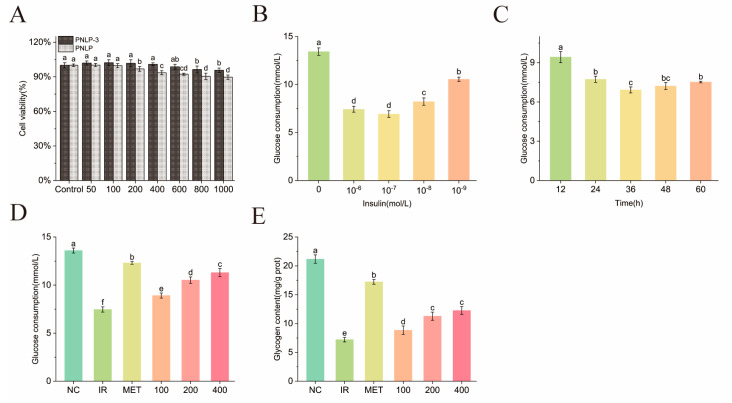
Effects of PNLP and PNLP-3 on the viability of HepG2 cells (**A**); effects of different insulin concentrations (**B**) and different time points (**C**) on glucose consumption in HepG2 cells; effects of PNLP-3 on glucose consumption (**D**) and glycogen synthesis (**E**) in IR-HepG2 cells. Note: The different superscript letters indicate a significant difference (*p* < 0.05) within the row based on Duncan’s test.

The MTT assay was employed to assess the cytotoxic effects of PNLP and PNLP-3 on HepG2 cells. As depicted in Figure 7A, neither PNLP nor PNLP-3 significantly affected the viability of HepG2 cells at lower concentrations. However, as the polysaccharide concentration increased, cell viability progressively declined. At a PNLP concentration of 200 μg/mL, the cell survival rate was 96.85%, which was significantly lower than the survival rate of 99.7% observed at 100 μg/mL (*p* < 0.05). The possible reason for this could be the presence of substances in PNLP that inhibit cell proliferation, such as certain phenolic compounds, alkaloids, and flavonoids [38]. PNLP-3 demonstrated no significant cytotoxicity to the cells across the concentration range of 0–600 μg/mL (*p* > 0.05); therefore, PNLP-3 was chosen for the subsequent hypoglycemic test using HepG2 cells.

(2) Establishment of IR-HepG2 Cell Models

Insulin resistance (IR) is a hallmark of type 2 diabetes mellitus (T2DM), characterized by reduced sensitivity and responsiveness to insulin in peripheral tissues such as the liver and muscles, ultimately leading to dysregulation of blood glucose levels [39]. HepG2 cells, which have high-affinity insulin receptors on their surface and exhibit a phenotype and function similar to normal liver cells, are an ideal model for studying diabetes and its complications. Figure 7B,C illustrate the impact of varying insulin concentrations and induction durations on glucose consumption in HepG2 cells. The lowest glucose consumption, measured at 6.92 ± 0.34 mmol/L, occurred when the insulin concentration was 10^−7^ mol/L and the induction period was 36 h. At this point, HepG2 cells exhibited diminished sensitivity to insulin, impairing their ability to effectively take up and utilize glucose. Consequently, an IR-HepG2 cell model was established by exposing HepG2 cells to 10^−7^ mol/L insulin for 36 h.

(3) Effects of PNLP-3 on Glucose Consumption and Glycogen Synthesis in IR-HepG2 Cells

Polysaccharides can alleviate the reduction in cell glucose uptake and utilization efficiency caused by insulin resistance and increase glycogen synthesis [40]. As shown in Figure 7D,E, the model group exhibited glucose consumption and glycogen synthesis levels of 7.47 ± 0.27 mmol/L and 7.21 ± 0.39 mg/g protein, respectively. These values were reduced by 0.82-fold and 1.94-fold, respectively, when compared to the normal group, indicating the onset of insulin resistance in HepG2 cells. Compared to the model group, both the metformin and polysaccharide treatment groups significantly enhanced glucose consumption and glycogen synthesis in IR-HepG2 cells, with the polysaccharide group demonstrating a dose-dependent response. At a PNLP-3 concentration of 400 μg/mL, glucose consumption and glycogen synthesis were elevated to 1.51-fold and 1.70-fold, respectively, relative to the model group. The results indicate that PNLP-3 effectively alleviates insulin resistance in HepG2 cells, promotes glucose uptake and utilization, and enhances glycogen synthesis, showing potential hypoglycemic effects. After PNLP-3 treatment, the increase in glucose consumption and glycogen synthesis in IR-HepG2 cells may be associated with the regulation of inflammatory pathways, activation of glycogen synthase, and enhanced expression of glucose transporters. Chen et al. [41] demonstrated that *Astragalus membranaceus* polysaccharides can regulate blood glucose by alleviating oxidative stress and liver damage. Yang et al. [42] showed that *Pumpkin* polysaccharides can alleviate hyperglycemia symptoms by modulating metabolites related to oxidative stress. Similarly, Ren et al. [42] reported that *Sargassum thunbergii* polysaccharides significantly enhance glucose uptake in IR-HepG2 cells, indicating that plant-derived polysaccharides show great potential in blood glucose regulation. Similarly, Ren et al. [43] reported that polysaccharides from *Sargassum fusiforme* significantly enhance glucose uptake in IR-HepG2 cells, highlighting the great potential of plant-derived polysaccharides in hypoglycemic effects. Therefore, further studies are needed to explore the underlying mechanisms of PNLP-3 in lowering blood glucose.

#### 2.6.3. Effects of PNLP-3 on the Antioxidant Levels in IR-HepG2 Cells

**Figure 8:**
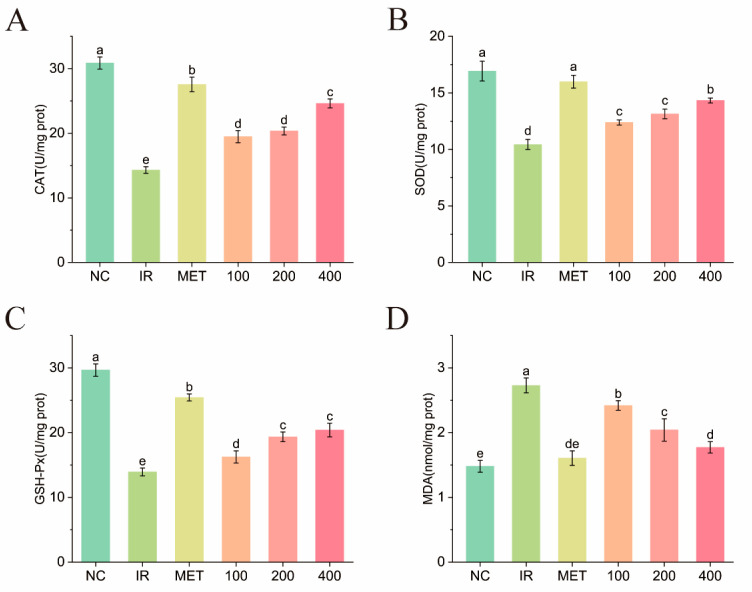
Effects of PNLP-3 on the antioxidant capacity of IR-HepG2 cells. (**A**) CAT enzyme activity; (**B**) SOD enzyme activity; (**C**) GSH-Px enzyme activity; (**D**) MDA content. Note: The different superscript letters indicate a significant difference (*p* < 0.05) within the row based on Duncan’s test.

In a high-oxidative-stress environment, disturbances in glucose metabolism can occur, further accelerating the progression of diabetes [44]. Endogenous antioxidant enzymes in the body, such as superoxide dismutase (SOD), catalase (CAT), and glutathione peroxidase (GSH-Px), constitute the first line of defense against free radical damage and play a crucial role in preventing oxidative injury. Malondialdehyde (MDA), a byproduct of lipid peroxidation, serves as a key indicator for assessing the extent of oxidative damage in the body. As depicted in Figure 8, the insulin resistance group exhibited a significant decrease in the activities of CAT, SOD, and GSH-Px (*p* < 0.05), while the MDA content was notably elevated (*p* < 0.05) when compared to the blank group. This indicates that under insulin resistance conditions, cells experience oxidative damage, leading to a decrease in antioxidant enzyme activity and severe lipid peroxidation. Following treatment with varying concentrations of PNLP-3, a significant dose-dependent increase in the activities of CAT, SOD, and GSH-Px was observed in IR-HepG2 cells, accompanied by a reduction in MDA content. However, this effect was less pronounced compared to the metformin group. Consequently, PNLP-3 appears to mitigate free radical damage by boosting the activity of antioxidant enzymes and suppressing lipid peroxidation in cells, thus reducing the oxidative stress in IR-HepG2 cells. This is consistent with the findings reported by Xiao et al. [45] and Wang et al. [46], who demonstrated that plant-extracted polysaccharides can alleviate glucose metabolism disorders by enhancing cellular antioxidant capacity.

In summary, PNLP-3 exhibits no significant cytotoxicity within the concentration range of 0–400 μg/mL (*p* < 0.05). Furthermore, it alleviated oxidative stress in IR-HepG2 cells and reduced insulin resistance by modulating glucose absorption, utilization, and glycogen synthesis, demonstrating a certain degree of hypoglycemic activity. This study has confirmed that PNLP-3 possesses in vitro hypoglycemic activity. The next step will be to conduct further research on the in vivo hypoglycemic mechanism of PNLP-3.

## 3. Materials and Methods

### 3.1. Materials and Reagents

*Panax notoginseng* leaves were purchased from Wenshan Panax Notoginseng Technology Industry Co., Ltd. (Wenshan, China). Cellulase and pectinase were purchased from Shanghai Macklin Biochemical Technology Co., Ltd. (Shanghai, China). α-glucosidase, α-amylase, and bovine insulin were purchased from YuanYe Biotechnology Co., Ltd. (Shanghai, China). HepG2 cells were obtained from the Kunming Cell Bank of the Chinese Academy of Sciences (Kunming, China). AB-8 macroporous adsorption resin, glucose, glycogen, SOD, CAT, GSH-Px, and MDA assay kits were purchased from Beijing Solarbio Technology Co., Ltd. (Beijing, China). Other reagents (analytical grade) were purchased from Sinopharm Chemical Reagent Co., Ltd. (Beijing, China).

### 3.2. Extraction of PNLP

PNLP extraction was performed following the methods reported by Chu et al. [14] and Liang et al. [47]. First, the powder of *Panax notoginseng* leaves was mixed with 95% ethanol (1:15, *w*/*v*) and heated under reflux at 50 °C for 3 h in a water bath to remove lipids, pigments, and low-molecular-weight impurities. The resulting residue was then collected and dried. Next, 50 g of the defatted powder was weighed and combined with deionized water at a 1:25 (*w*/*v*) ratio, with the pH adjusted accordingly. A 2% composite enzyme solution, consisting of cellulase and pectinase in equal proportions, was added to the mixture. This was then subjected to ultrasonic extraction at 50 °C and 300 W for 60 min to extract the polysaccharides. Following extraction, the mixture was heated for 5 min to deactivate the enzymes, and the supernatant was separated by centrifugation. After vacuum concentration to one-quarter of the original volume, proteins were removed using Sevag reagent, followed by decolorization with AB-8 macroporous resin. The filtrate was collected and subjected to alcohol precipitation overnight at 4 °C. The precipitate was then collected and freeze-dried to obtain PNLP.

### 3.3. Single-Factor Experiment and Response Surface Optimization

Through the single-factor experiment, the effects of seven factors on the yield of PNLP were preliminarily investigated: pH of the extraction solution (4.0, 4.5, 5.0, 5.5, and 6.0), material/liquid ratio (1:15, 1:20, 1:25, 1:30, and 1:35 g/mL), cellulase/pectinase ratio (1:3, 1:2, 1:1, 2:1, and 3:1), enzyme addition amount (1.0%, 1.5%, 2.0%, 2.5%, and 3.0%), ultrasound time (20, 40, 60, 80, and 100 min), ultrasound power (100, 200, 300, 400, and 500 W), and ultrasound temperature (30, 40, 50, 60, and 70 °C).

Based on the findings from the single-factor experiment, four factors with significant effects namely pH (*A*), material/liquid ratio (*B*), enzyme addition amount (*C*), and ultrasound time (*D*), were selected as independent variables. The extraction yield of PNLP was taken as the response variable. A response surface optimization experiment was subsequently designed, with the experimental factors and their respective levels outlined in Table 4.

### 3.4. Separation and Purification of PNLP

PNLP was isolated and purified following the methods reported by Zhang et al. [48] and Li et al. [49]. A 10 mg/mL polysaccharide solution was slowly applied along the wall of a DEAE-Cellulose 52 column (26 mm × 50 cm). The sample was then sequentially eluted with distilled water and NaCl solutions of concentrations 0.1, 0.3, and 0.5 M. The elution was conducted at a flow rate of 0.5 mL/min, with the eluate collected in 12 min intervals per tube. Absorbance was quantified at 490 nm using the phenol-sulfuric acid assay, and the resulting elution profile was subsequently plotted. Based on the elution profile, four distinct fractions were obtained: PNLP-1 (eluted with distilled water), PNLP-2 (eluted with 0.1 M NaCl), PNLP-3 (eluted with 0.3 M NaCl), and PNLP-4 (eluted with 0.5 M NaCl).

### 3.5. Molecular Composition and Structural Characterization

#### 3.5.1. Chemical Composition

The total sugar content of PNLP and PNLP-3 was quantified using the phenol-sulfuric acid method [50], with a standard curve equation of y = 9.8325x + 0.0663 and *R*^2^ = 0.9992. Protein content was assessed via the Bradford method, utilizing a standard curve equation of y = 0.6386x + 0.1538 and *R*^2^ = 0.9991. The uronic acid content was measured using the carbazole-sulfuric acid method, with glucuronic acid serving as the standard [51], yielding a standard curve equation of y = 0.696x + 0.0907 and *R*^2^ = 0.9985.

#### 3.5.2. Monosaccharide Composition

Eleven monosaccharide standards (fucose, rhamnose, arabinose, galactose, glucose, xylose, mannose, fructose, ribose, galacturonic acid, and glucuronic acid) and 5 mg of sample each were precisely weighed and placed into ampoules, followed by the addition of 2 mL of 3 M TFA. The samples were hydrolyzed at 120 °C for 3 h and then dried under nitrogen. Subsequently, 5 mL of distilled water was added and mixed by vortexing. The mixture was centrifuged at 12,000 rpm for 5 min, and the supernatant was collected for analysis using an ion chromatograph (ICS5000, ThermoFisher, Waltham, MA, USA) [52]. The chromatographic conditions were as follows: column: Dionex Carbopac^TM^ PA10 (4 × 250 mm); mobile phases: A: H_2_O; B: 500 mM NaOH and 50 mM NaOAc; C: 20 mM NaOH; flow rate: 1.0 mL/min; injection volume: 25 µL; column temperature: 30 °C; detector: electrochemical detector.

#### 3.5.3. Mw

Each sample was dissolved in the mobile phase to prepare a 5 mg/mL solution. After centrifugation and filtration, the solution was analyzed by HPGPC [53]. The chromatographic conditions were as follows: the mobile phase consisted of 0.05 M NaCl solution; the column used was BRT 105-103-101 (8 × 300 mm, Beijing, China); the flow rate was set to 0.7 mL/min; the column temperature was maintained at 40 °C; the injection volume was 25 μL; and detection was performed using a differential refractive index detector (RI-10A, Shimadzu, Tokyo, Japan). A standard curve was generated using pullulan as the reference, and the molecular weights of PNLP and PNLP-3 were determined based on the retention times of the samples.

#### 3.5.4. FT-IR

The functional groups of PNLP and PNLP-3 were characterized using FT-IR (IRTracer, Shimadzu, Japan). The dried polysaccharides were carefully mixed and ground with KBr powder before being placed into the IR spectrometer for analysis. Scanning was performed within the range of 400–4000 cm^−1^ with a resolution of 4 cm^−1^, and the resulting infrared absorption spectra were subsequently analyzed.

#### 3.5.5. SEM

The dried PNLP and PNLP-3 were placed on a sample stage, gold-coated, and then observed under a scanning electron microscope (SEM 5000×, Guoyi Quantum, Hefei, China). The operating conditions were as follows: accelerating voltage of 15 kV, with magnifications of 500× and 2000×. Representative images were taken for documentation.

### 3.6. Antioxidant Activity Measurement

The antioxidant activity of PNLP and PNLP-3 was assessed following the protocols outlined by Zhu et al. [54] and Wang et al. [55]. Solutions of the polysaccharides at varying concentrations (0.25, 0.5, 1, 2, 3, 4, and 5 mg/mL) were prepared, and then DPPH, ABTS, hydroxyl, and superoxide anion radical scavenging abilities were determined, respectively, with Vitamin C (Vc) as a positive control.

### 3.7. In Vitro Hypoglycemic Activity Assay

#### 3.7.1. Digestive Enzyme Activity Inhibition Analysis

Enzyme activity inhibition assays for α-glucosidase and α-amylase were conducted following the protocols outlined by Yang et al. [33] and Hu et al. [28], with minor modifications. Solutions of PNLP and PNLP-3 at varying concentrations (0.25, 0.5, 1, 2, 4, and 8 mg/mL) were prepared in a phosphate buffer (pH = 6.9), and acarbose was used as the positive control in all experiments.

Molecular-Docking-Based Enzyme Activity Inhibition Potential Analysis: The three-dimensional structures of α-glucosidase (ID: P53341) and α-amylase (ID: Q6PMJ3) were obtained from the UniProt database and were subjected to hydrogenation and charge treatment using AutoDock-1.5.6. The main monosaccharide components of PNLP-3, arabinose and galactose, were selected for molecular docking. The Affinity value (kcal/mol) indicates the strength of the binding interaction between the ligand and receptor; a lower Affinity value signifies a more stable binding. The results were visualized using Discovery Studio 2019.

#### 3.7.2. Study on the Hypoglycemic Effect of PNLP-3 in Regulating IR-HepG2 Cells

Cell Viability Assay: The MTT assay method was performed with slight modifications based on the protocol reported by Hao et al. [56]. HepG2 cells were seeded in a 96-well plate at a density of 1 × 10^4^ cells per well and cultured for 12 h. The culture medium was then discarded after the cells had adhered. The cells were then treated with varying concentrations (50, 100, 200, 400, 600, 800, 1000 μg/mL) of PNLP and PNLP-3 solutions, and incubation continued for 24 h. Then, 5 mg/mL MTT solution was added, and the cells were cultured for 4 h. Following incubation, 150 μL of DMSO was added to each well to stop the reaction, and the plate was gently shaken for 10 min. The absorbance at 570 nm was measured for each well, and cell viability was calculated using the appropriate formula, as follows:

HepG2 Cell Viability (%) = (A_s_ − A_b_)/(A_c_ − A_b_) × 100%

where A_s_ denotes the absorbance of the experimental group, A_b_ represents the absorbance of the blank group (no cells), and A_c_ indicates the absorbance of the control group.

Establishment of the Insulin Resistance HepG2 (IR-HepG2) Cell Model: The model was established following the method reported by Zheng et al. [57]. HepG2 cells were seeded in a 6-well plate at a density of 1 × 10^6^ cells per well and cultured for 12 h until adhesion, after which the culture medium was discarded. Then, DMEM high-glucose medium containing different concentrations of insulin (0, 10^−6^, 10^−7^, 10^−8^, 10^−9^ mol/L) was added, and the cells were cultured for different periods (12, 24, 36, 48, 60 h). Finally, glucose content in the supernatant was measured using a kit, and the glucose inhibition rate was calculated to determine the optimal conditions for insulin action.

Glucose Consumption and Glycogen Content Assay: The IR-HepG2 cell model was established, with groups designated as control (NC), model (IR), positive (MET), and polysaccharide protection. After 24 h of incubation, glucose levels in the supernatant and glycogen content in the cell pellet were assessed using appropriate assay kits. The control group comprised normal cells, while the model group included IR-HepG2 cells. The polysaccharide protection group was treated with varying concentrations of PNLP-3 (100, 200, 300 μg/mL) in DMEM high-glucose medium, and the positive control group was treated with metformin (100 μg/mL).

#### 3.7.3. Determination of Antioxidant Levels

HepG2 cells were plated at a density of 2 × 10⁶ cells per well in 60 mm culture dishes and incubated for 12 h to facilitate adhesion. The IR-HepG2 cell model was subsequently established, with groups designated as control, model, polysaccharide protection, and positive. Following various treatments, proteins were extracted by ice-cold ultrasonic disruption, and the levels of CAT, SOD, GSH-Px, and MDA in the cells were measured using commercial kits.

### 3.8. Statistical Analysis

Graphical processing was performed using Origin 2018 and Design-Expert 13.0. Data analysis was conducted using SPSS 19.0 software with Duncan’s multiple range test. All experiments were repeated three times, and the data are presented as mean ± standard deviation. *p* < 0.05 was considered to indicate statistical significance.

## 4. Conclusions

In summary, this study optimized the process conditions for the ultrasound-assisted dual-enzyme extraction of PNLP through a single-factor combined with response surface experiment, achieving an extraction yield of 17.13 ± 0.29%. The purified component PNLP-3, isolated by DEAE-Cellulose 52, had a total sugar content of 81.2% and a molecular weight of 16.57 kDa. PNLP-3 is primarily composed of arabinose and galactose, with β-glycosidic bonds in a pyranose form. Additionally, PNLP-3 exhibits good antioxidant activity, as well as inhibitory activities against α-glucosidase and α-amylase. Molecular docking results indicated that the polysaccharide shows stronger inhibitory activity against α-glucosidase. In vitro cell experiments confirmed that PNLP-3 promotes glucose consumption and glycogen synthesis, alleviates oxidative stress in IR-HepG2 cells, and demonstrates good hypoglycemic potential. This study provides a theoretical basis for the efficient extraction and resource utilization of PNLP, as well as references for further exploring the in vivo hypoglycemic activity of PNLP-3.

## Figures and Tables

**Table 1:** Response surface experiment design and results.

Number	*A* pH	*B* Ratio of Material to Water (g/mL)	*C* Compound Enzyme Concentration (%)	*D* Ultrasonic Time (min)	*Y* Extraction Yield (%)
1	5.0	25	2.0	60	17.21 ± 0.22
2	5.0	25	2.0	60	17.01 ± 0.23
3	5.0	20	2.0	40	15.56 ± 0.16
4	5.0	25	2.0	60	17.30 ± 0.35
5	5.0	25	1.5	80	15.80 ± 0.21
6	5.0	30	2.0	40	15.71 ± 0.29
7	5.0	25	1.5	40	15.30 ± 0.21
8	5.0	25	2.5	40	16.92 ± 0.14
9	4.5	25	2.0	80	16.40 ± 0.29
10	5.0	30	2.5	60	16.51 ± 0.16
11	5.0	25	2.0	60	17.30 ± 0.24
12	5.5	25	2.5	60	17.20 ± 0.14
13	5.5	30	2.0	60	16.41 ± 0.20
14	5.5	25	2.0	80	17.07 ± 0.11
15	4.5	25	2.0	40	16.04 ± 0.32
16	4.5	20	2.0	60	15.51 ± 0.28
17	5.0	30	1.5	60	15.71 ± 0.37
18	4.5	25	1.5	60	15.40 ± 0.26
19	5.5	25	2.0	40	15.60 ± 0.15
20	5.0	20	2.5	60	15.81 ± 0.10
21	5.0	30	2.0	80	16.21 ± 0.31
22	5.0	25	2.0	60	17.07 ± 0.28
23	5.0	20	1.5	60	14.70 ± 0.34
24	5.0	20	2.0	80	15.51 ± 0.25
25	5.5	20	2.0	60	15.40 ± 0.19
26	5.0	25	2.5	80	17.25 ± 0.14
27	4.5	30	2.0	60	16.20 ± 0.26
28	5.5	25	1.5	60	15.22 ± 0.17
29	4.5	25	2.5	60	16.40 ± 0.14

Note: Mean ± SD, *n* = 3.

**Table 2:** ANOVA of response surface test results.

Source	Sum of Squares	df	Mean Square	*F* Value	*p* Value Prob > F	
Model	15.42	14	1.10	18.11	<0.0001	significant
*A*-pH	0.0744	1	0.0744	1.22	0.2874	
*B*-Ratio of material to water(g/mL)	1.51	1	1.51	24.86	0.0002	**
*C*-Compound enzyme concentration (%)	5.27	1	5.27	86.57	<0.0001	**
*D*-Ultrasonic time(min)	0.8124	1	0.8124	13.36	0.0026	**
*AB*	0.0254	1	0.0254	0.4174	0.5287	
*AC*	0.2422	1	0.2422	3.98	0.0658	
*AD*	0.2998	1	0.2998	4.93	0.0434	*
*BC*	0.0246	1	0.0246	0.4042	0.5352	
*BD*	0.0736	1	0.0736	1.21	0.2900	
*CD*	0.0069	1	0.0069	0.1134	0.7413	
*A* ^2^	1.46	1	1.46	24.04	0.0002	**
*B* ^2^	5.58	1	5.58	91.74	<0.0001	**
*C* ^2^	2.00	1	2.00	32.86	<0.0001	**
*D* ^2^	1.10	1	1.10	18.06	0.0008	**
Residual	0.8516	14	0.0608			
Lack of Fit	0.7790	10	0.0779	4.29	0.0865	not significant
Pure Error	0.0726	4	0.0181			
Cor Total	16.28	28				

Note: *p* < 0.05 *; *p* < 0.01 **.

**Table 3:** Yields and Chemical Compositions of PNLP, PNLP-1, PNLP-2, PNLP-3, and PNLP-4.

Indicator	PNLP	PNLP-1	PNLP-2	PNLP-3	PNLP-4
Yield (%)	17.13 ± 0.29	0.78 ± 0.16	2.69 ± 0.24	5.64 ± 0.21	1.04 ± 0.14
Total sugar content (%)	24.68 ± 0.93	59.53 ± 0.85	70.31 ± 0.85	81.20 ± 1.17	72.52 ± 1.08
Protein content (%)	6.15 ± 0.21	3.19 ± 0.16	2.62 ± 0.13	1.25 ± 0.17	1.4 ± 0.13
Uronic acid content (%)	7.75 ± 0.33	10.58 ± 0.22	12.5 ± 0.44	16.33 ± 0.30	9.09 ± 0.38

Note: Mean ± SD, *n* = 3.

**Table 4:** Level and code of independent variables used for response surface analysis.

Factor	Level		
	−1	0	1
*A* pH	4.5	5.0	5.5
*B* Ratio of material to water (g/mL)	1:20	1:25	1:30
*C* Compound enzyme concentration (%)	1.5	2.0	2.5
*D* Ultrasonic time (min)	40	60	80

## Data Availability

The authors confirm that the data supporting the findings of this study are available within the article.
